# Strengthening public health programmes and the antivenom ecosystem: strategic policy priorities to combat envenomation by animal bites and stings in Latin America and the Caribbean

**DOI:** 10.7189/jogh.16.04329

**Published:** 2026-10-09

**Authors:** María Elisa Peichoto, José María Gutiérrez, Hui Wen Fan, Camila Pinto Damasceno, Marco Antonio Natal Vigilato, the Latin American Network of Public Antivenom Manufacturing Laboratories (RELAPA) Collaboration, the Latin American Network of National Programs for Venomous Animal Accidents (REDPEVA) Collaboration

**Affiliations:** 1 Pan American Center for Foot-and-Mouth Disease and Veterinary Public Health, Pan American Health Organization, Duque de Caxias, Brazil; 2 Instituto de Biología Subtropical, Consejo Nacional de Investigaciones Científicas y Técnicas-Universidad Nacional de Misiones, Puerto Iguazú, Argentina; 3 Instituto Clodomiro Picado, Facultad de Microbiología, Universidad de Costa Rica, San José, Costa Rica; 4 Instituto Butantan, São Paulo, Brazil

**Keywords:** accidents by venomous animals, antivenom production and accessibility, epidemiological profile and trends, health system capacity, public health preparedness and response

## Abstract

**Background:**

Envenomation by snakes, scorpions, spiders, and caterpillars remains a major but unevenly addressed public health challenge in Latin America and the Caribbean (LAC). Environmental change, urbanisation, and ecological disruption are changing the distribution of medically important species, while persistent gaps in surveillance, clinical management, and antivenom availability limit prevention and care. In this study, we analysed epidemiological trends, health system capacity, and the regional antivenom ecosystem to inform strategic public health priorities.

**Methods:**

We analysed secondary data from regional surveys coordinated by Pan American Health Organization networks for the period 2019–2024. We used negative binomial regression models to assess temporal trends in total cases and deaths from four major envenomations and to generate projections up to 2030. We used spatial analyses to examine heterogeneity in incidence, mortality, and case-fatality rates, and evaluated antivenom production, procurement, and use with indicators standardised to epidemiological burden. We complemented the quantitative findings with qualitative content analysis of regional and meeting reports.

**Results:**

The incidence of all envenomations showed an increasing trend over time, with particularly marked increases in spider bites and caterpillar contacts. We observed spatial heterogeneity across countries. Overall mortality remained low for most envenomations, while the case-fatality rate for spider envenomation was projected to increase by 2030 if the observed trends continued. We identified weaknesses in surveillance quality, risk mapping, clinical preparedness, and follow-up of sequelae. We also identified mismatches between antivenom production, procurement, and therapeutic use, varying by envenomation type. Regional initiatives have strengthened coordination but have not fully resolved inequities in access and preparedness.

**Conclusions:**

Envenomation in LAC remains an expanding and evolving public health challenge requiring coordinated, forward-looking responses. While acknowledging limitations in data quality and the need for longer-term evidence, our findings provide a system-level analysis and generate scientifically and policy-relevant evidence, supporting a targeted action framework to advance progress toward the World Health Organization 2030 targets for reducing envenomation-related morbidity and mortality.

Envenomation by venomous animals (EVAs) constitutes a severe global public health problem, responsible for high mortality, morbidity, and permanent disabilities worldwide. The highest impact is caused by envenomation by snakes [1], scorpions [2] and spiders [3], although cases by other animal species are also frequent in various regions of the world. Snakebite envenomation (SBE) causes more than 100,000 deaths every year and leaves a high number of people with permanent physical and psychological sequelae [1]. It mainly affects impoverished rural populations in sub-Saharan Africa, Asia and Latin America, and was included in the list of neglected tropical diseases of the World Health Organization (WHO) in 2017 [4]. The WHO issued a global strategy targeting a 50% reduction in mortality and disability due to SBE by 2030 [5].

Latin America and the Caribbean (LAC) is the third most affected region by SBE (after Asia and Africa), reporting nearly 60,000 snakebite cases annually, although the actual numbers are likely to be higher owing to underreporting in remote rural areas. The region also has a high incidence of envenomations from scorpion stings, spider bites, and contact with *Lonomia* spp. caterpillars [6]. Moreover, some of these EVAs, such as those involving scorpions [7] and *Lonomia* spp. caterpillars [8], are emerging as a consequence of multiple factors, including climate change, anthropogenic processes impacting the environment, changes in land use, and the high ecological versatility of some species. These factors are altering the distribution and behavioural patterns of some venomous species, including the growth of several scorpion species of the genus *Tityus* in South America [9], and/or urban expansion that directly drives the invasion into venomous species’ natural habitats. Thus, addressing EVAs in LAC requires effective and integrated interventions.

LAC has a long tradition in the development of public health policies and regional cooperation programmes related to EVAs. Regional training courses were organised at Instituto Butantan (São Paulo, Brazil) during the 2000s; a network of public antivenom manufacturing laboratories in the region was established [10], and a regional consultation organised by the Pan American Health Organization (PAHO) was held in Brazil in 2007. After a resolution on SBE was adopted by the World Health Assembly in 2018 [4], PAHO started coordinating regional activities related to EVAs through its Pan American Center for Foot-and-Mouth Disease and Veterinary Public Health (PANAFTOSA). The Latin American Network of Public Antivenom Manufacturing Laboratories (RELAPA) was established in 2018 [11], and since then, two strategic planning meetings (the last held in May 2025) [12] and other related initiatives have taken place [13], allowing coordinated progress towards significant improvements in the design and production of antivenoms, which have impacted their efficacy and safety [14].

PANAFTOSA/PAHO has also coordinated a regional network involving representatives from ministries of health responsible for EVA programmes, which led to the creation of the Latin American Network of National Programs for Venomous Animal Accidents (REDPEVA) at its first in-person meeting held in Brazil in August 2025 [15]. PANAFTOSA/PAHO has conducted and consolidated multiple regional surveys targeting public antivenom manufacturers and ministries of health to generate evidence to inform decision-making [6,16,17]. Despite these efforts, important knowledge gaps persist, including the lack of integrated analyses linking epidemiological burden with health system capacity and antivenom availability, as well as a limited understanding of temporal trends and future projections of envenomation. To address these gaps, we provide a system-level assessment of the four major envenomations and related health system responses at the regional scale, generating comparative, policy-relevant evidence.

## METHODS

### Data sources and study design

We retrieved data on total cases and deaths from the four main types of venomous animal accidents (snakebites, scorpion stings, spider bites and contacts with caterpillars) from REDPEVA surveys [6,16]. As this is a reanalysis of these existing data sets, we confirm that our study adheres to the Journal of Global Health’s GRABDROP guidelines (Table S1 in the **Online Supplementary Document**) [18].

### Temporal trend analysis and projections

We fitted negative binomial regression models to evaluate the spatial epidemiology and temporal trends of EVAs in LAC for 2019–2024. Regression models are frequently used in epidemiological modelling studies to analyse temporal trends and predict the burden of these events [19]. We based data interpretation primarily on the effect of time, focusing on data analysis per year. For each EVA category and year, we aggregated country-level observations by summing the number of reported cases, deaths, and population [20] across Latin American countries, generating one annual regional total per type of envenoming. We selected negative binomial regression because the outcome variables consisted of count data with evidence of overdispersion relative to the Poisson distribution [21]. We fitted models using a log link function and the negative binomial regression (NB2) parameterisation. We centred the year variable on 2019 to improve parameter interpretability and numerical stability.

For each type of envenoming, we fitted two independent models: one for total cases and one for total deaths. We included the logarithm of the total regional population as an offset in both models, allowing temporal trends to be interpreted as changes in population-adjusted rates.

We used the formula *Y_t_^cases^* ~ NB2(*μ_t_*, *θ*); log (*μ_t_*) = (*β*_0_ + *β*_1_(*year_t_*) + log (*population_t_*) for the total case model and *Y_t_^cases^* ~ NB2(*μ_t_*, *θ*); log (*μ_t_*) = (*β*_0_ + *β*_1_(*year_t_*) + log (*population_t_*) for the total death model. Here, (*t*) *Y_t_^cases^* represents the total number of cases and *Y_t_^deaths^* the total number of deaths, both observed in Latin America in year *t*; *μ_t_* stands for the expected number of cases; *θ* for the dispersion parameter of the negative binomial distribution; *β*_0_ for the intercept; *β*_0_ for the coefficient associated with the temporal trend; and log (*population_t_*) for the offset corresponding to the total population of Latin America in year *t*.

We generated illustrative projections for 2025–2030 using the fitted negative binomial models in combination with United Nations population projections. These projections were intended to explore the expected regional trajectory under the assumption that the average temporal trend observed during the study period would remain unchanged, rather than to provide precise epidemiological forecasts. We estimated standard errors on the link scale and calculated 95% confidence intervals (CIs) using the normal approximation and subsequently back-transformed to the response scale by exponentiation. Given the limited number of annual observations available for model fitting, the projected confidence intervals should be interpreted as reflecting model uncertainty under the assumed trend rather than precise estimates of future epidemiological behaviour.

The objective of the regression analysis was to illustrate regional temporal trends for Latin America. Therefore, we aggregated country-level observations into annual regional totals prior to model fitting. Consequently, we did not explicitly model between-country variability, as the unit of inference was the regional aggregate rather than individual countries.

### Spatial epidemiological analysis

We estimated the epidemiological metrics of incidence (per 100,000 inhabitants), mortality (per 100,000 inhabitants), and fatality rate (%) based on the model illustrations, using annual averages for the 2019–2024 period. We categorised variables into global quantile classes calculated from the distribution of the selected metric across all countries and EVA types. We used this strategy to establish a comparable scale across panels. For SBE, we also calculated the difference between the predicted case-fatality rate for 2030 and those for 2019 to assess progress towards the WHO global goal of 50% reduction in deaths. We applied the same calculation to the other three envenomations included in the study, although no WHO global targets have been defined for them.

Furthermore, we used a shapefile of Latin American countries to represent the spatial epidemiology of EVAs. We carried out a literature search to extract data for 2019–2024 for countries without data from survey reports [22]. When no epidemiological data were available after consultation of REDPEVA reports and the published literature, we estimated values using the k-nearest neighbours methodology [23]. Where countries exhibited comparable geographic, ecological, and demographic characteristics, we derived estimates from the average values observed in the most similar neighbouring countries. We applied this procedure exclusively to Suriname; for all other countries lacking sufficiently comparable neighbours, we performed no imputation, and values remained unavailable for mapping purposes (Table S2 in the **Online Supplementary Document**).

### Assessment of the antivenom ecosystem

Considering that antivenoms are the only effective treatment for the deleterious clinical effects of EVAs, we applied the same modelling methodology, using a negative binomial distribution, to the following three core dimensions of the antivenom ecosystem: production in public laboratories (public structural capacity), procurement (demand and supply capacity), and use (treatment capacity). We considered antivenom categories for snakes, scorpions, spiders, and *Lonomia*.

We obtained production data from the RELAPA survey [17], referring to antivenom vials produced by 12 public laboratories in Latin America. Procurement and administration data reflect the supply of antivenoms by public or private laboratories, and their use within healthcare systems [6,16]. Antivenoms were categorised according to the type of envenomation they are intended to treat: snakebite, scorpion sting, spider bite, and caterpillar contact. Antivenom produced by the Butantan Institute (Brazil) for arachnid envenomation contains antibodies against the venoms of the spiders *Loxosceles gaucho* and *Phoneutria nigriventer*, as well as the scorpion *Tityus serrulatus*. As this study analyses spider and scorpion antivenoms separately, we proportionally allocated the total number of vials of this polyvalent antivenom, assigning 80% for scorpion envenomation and 20% for spider envenomation. This distribution is based on epidemiological data from Brazil [24] and reflects the fact that production is primarily tailored to meet domestic demand.

Additionally, we estimated the regional average number of vials recommended for treatment using the average number of vials required for each severity category (mild, moderate, and severe) and the average proportion of cases in each severity category, based on therapeutic guidelines [25–30] and previously reported data [8,31–43], respectively.

By comparing these data with the total number of antivenom vials produced, procured, or used, we aimed to assess the consistency between the three core dimensions of the antivenom ecosystem and the specific epidemiological dynamics of each type of envenomation.

### Qualitative content analysis

We performed qualitative content analysis of survey and meeting reports to obtain additional insights. Particular attention was given to identifying strengths, challenges, and priorities related to surveillance, clinical care, antivenom availability, and public health programme implementation. These findings were used to contextualise the quantitative analyses and inform the development of strategic recommendations for LAC.

### Statistical analysis

We conducted all analyses in *R*, version 4.3.3 (R Core Team, Vienna, Austria). We defined statistical significance as a *P* value <0.05. Parameter estimates are presented with their corresponding 95% CIs where applicable.

## RESULTS

### Epidemiological burden and spatial-temporal trends

Between 2019 and 2024, accidents involving venomous animals in LAC continued to represent a significant public health concern, characterised by a high frequency of cases and substantial variation in lethality depending on the species involved.

Scorpion stings represented the vast majority of reported events (2,850,408 cases; incidence 221.1 per 100,000 inhabitants), whereas snakebites were associated with the highest case-fatality rate (0.40%) despite a much lower incidence (24.2 per 100,000 inhabitants) (**Table 1**).

**Table 1:** Epidemiological profile of accidents caused by venomous animals in Latin America and the Caribbean (2019–2024)*

	Total number of cases	Total number of deaths			Incidence per 100,000 inhabitants	Mortality per 100,000 inhabitants	Case-fatality rate, %
**Snake bite**	312,470		1,250	24.24		0.097	0.400
**Scorpion sting**	2,850,408		850	221.10		0.066	0.030
**Spider bite**	349,419		334	27.10		0.026	0.096
**Caterpillar contact**	23,595		10	1.83		0.001	0.042
**Total**	3,535,892		2,444	274.27		0.190	0.069

*The population size considered was the average population over the six years of observation, totalling 1,289,167,799 people.

Regarding temporal trends, we observed a significant increase in the number of cases associated with snakebites (*β* = 0.032; *P* = 0.002), scorpion stings (*β* = 0.032; *P* = 0.022), spider bites (*β* = 0.147; *P* = 0.003), and contacts with caterpillars (*β* = 0.433; *P* = 0.0006), corresponding to an approximate annual increase of 3.3%, 3.3%, 15.8% and 54.2% per year, respectively (**Figure 1**; Table S3 in the **Online Supplementary Document**).

**Figure 1:**
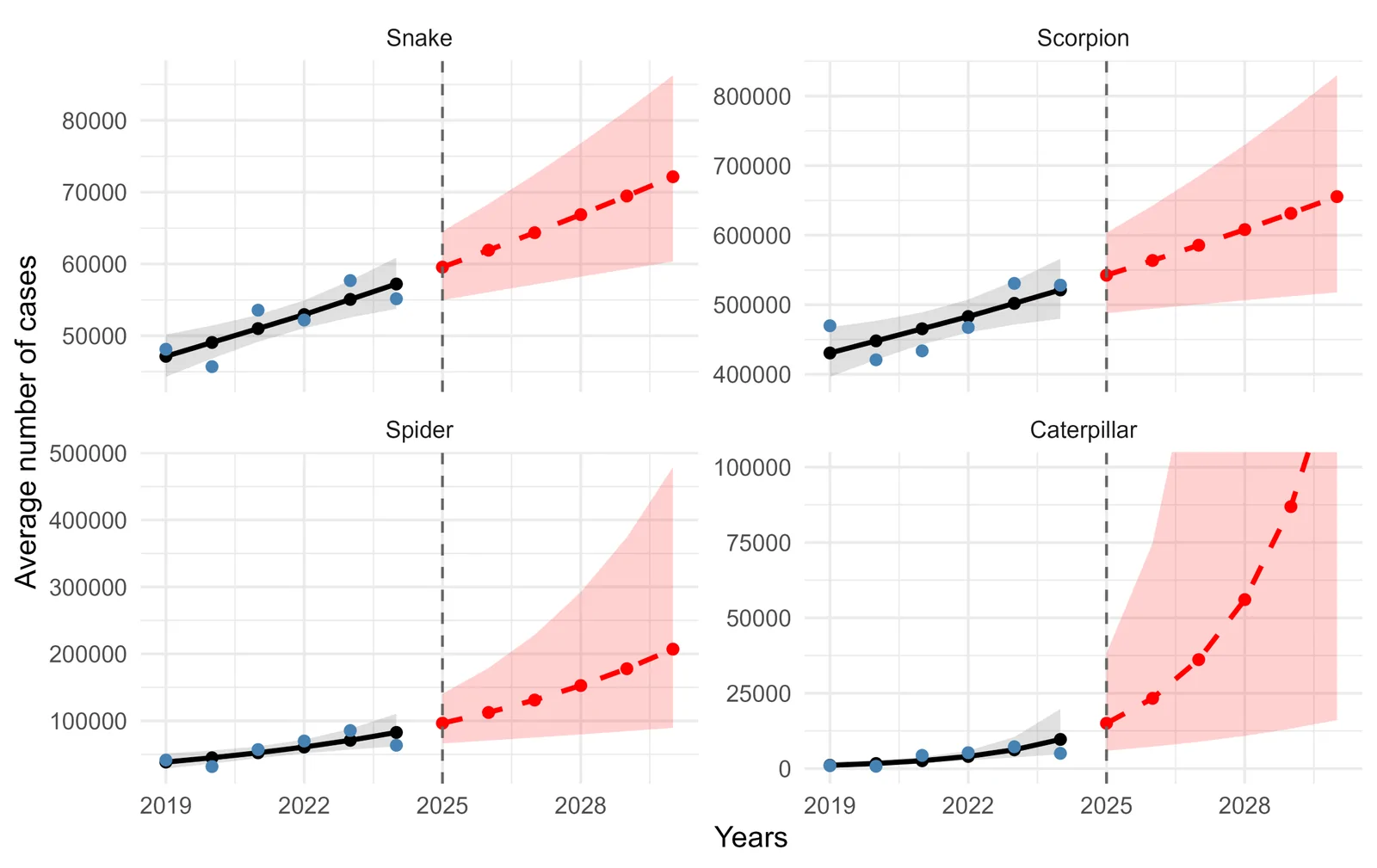
Predictions of the number of cases per type of venomous animal involved in the events. The panels show the trend in the annual regional number of cases over the observed period (2019–2024; solid black lines) and the predicted period (2025–2030; dashed red lines), estimated using negative binomial models with population compensation. The blue points represent the observed mean annual values. The shaded bands represent the 95% confidence intervals of the expected value (*μ*). The dotted vertical line indicates the start of the predicted period.

Spider-related accidents showed a significant increasing trend in the total regional deaths (*β* = 0.179; *P* < 0.001), corresponding to an estimated average annual growth of 19.6%. For the other EVAs, we observed no statistical evidence of a temporal trend (*P* > 0.05 in all cases), reflecting high variability and a low frequency of deaths in these series (**Figure 2**; Table S4 in the **Online Supplementary Document**).

**Figure 2:**
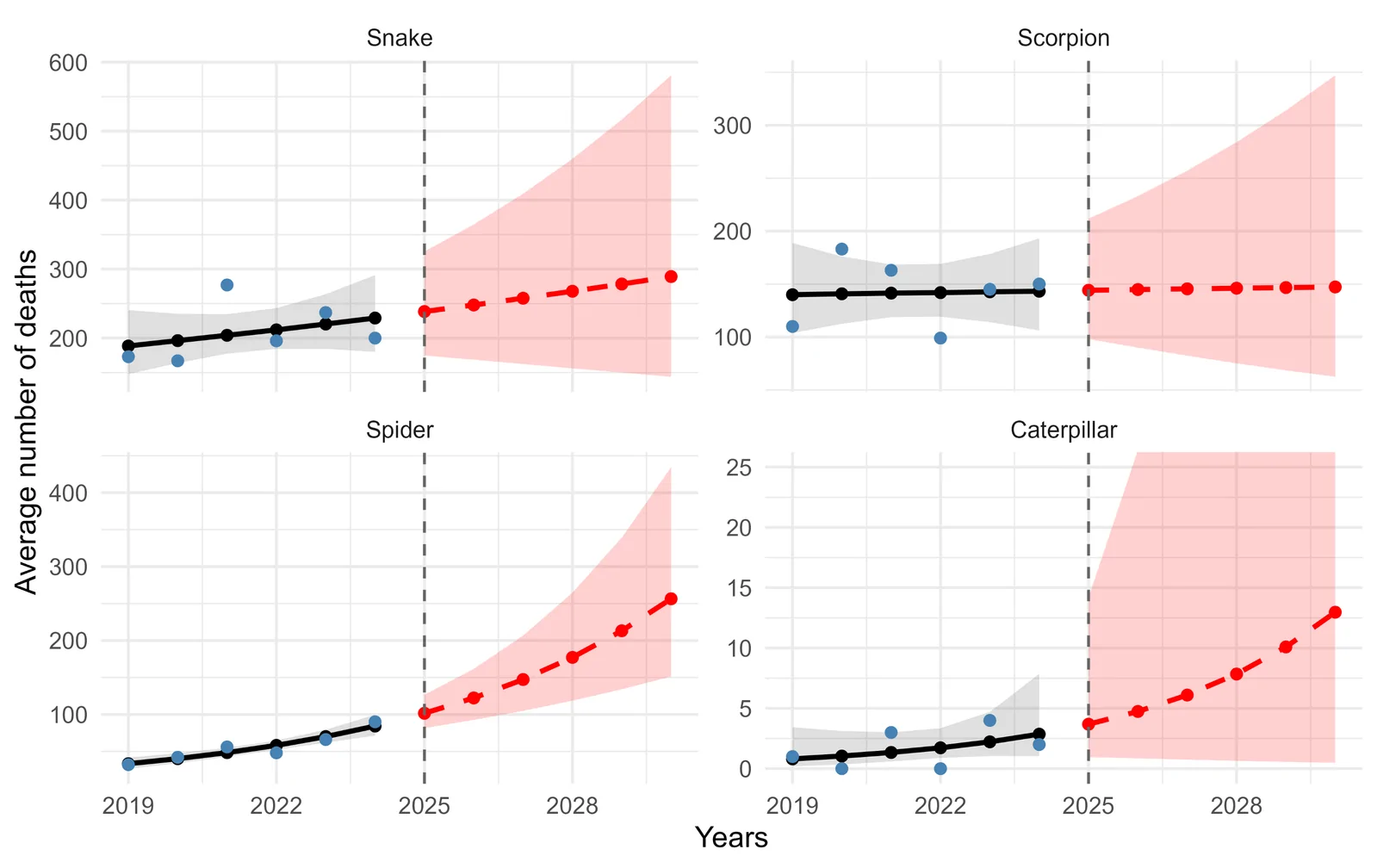
Predictions of the number of deaths per type of venomous animal involved in the events. The panels show the trend in the annual regional number of deaths over the observed period (2019–2024; solid black lines) and the predicted period (2025–2030; dashed red lines), estimated from negative binomial models with population compensation. The blue points represent the observed mean annual values. The shaded bands represent the 95% confidence intervals of the expected value (*μ*). The dashed vertical line indicates the start of the predicted period.

Based on the generated predictions, the case-fatality rate associated with snakebites was projected to remain essentially stable by 2030, showing only a 0.23% increase between 2019 and 2030. In contrast, substantial reductions in case fatality rates were projected for scorpion stings (−31%) and caterpillar contacts (−87%), whereas spider-related accidents were projected to experience a marked increase (+43%).

Across countries, snakebite incidence varied by more than 20-fold and case-fatality rates by more than 15-fold across countries, while scorpion sting incidence ranged from less than 1 to over 200 cases per 100,000 inhabitants. Spider-bite incidence also varied markedly across countries, ranging from less than 1 to almost 60 cases per 100,000 inhabitants, whereas caterpillar-related accidents were concentrated in relatively few countries and remained rare or absent throughout much of the region (**Figure 3**).

**Figure 3:**
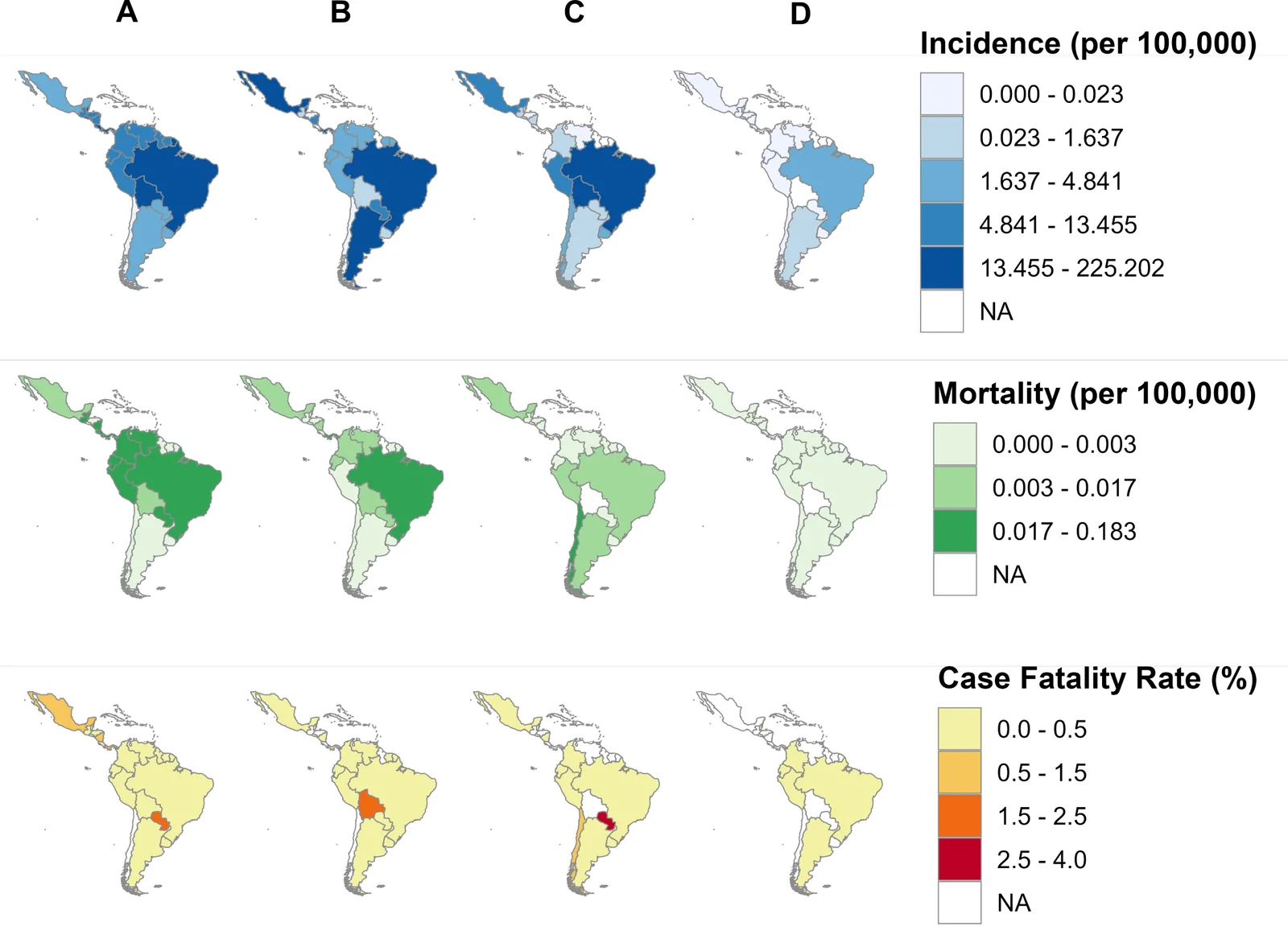
Spatial epidemiology of EVAs. **Panels A–D.** Snakebites, scorpion stings, spider bites, contacts with caterpillars in Latin America and the Caribbean. The maps show the average annual incidence (per 100,000 inhabitants), mortality (per 100,000 inhabitants) and fatality rate (%), calculated for each country over the period 2019–2024. The colours represent classes defined from the distribution of modelled values.

Overall, snakebites and scorpion stings display the highest and most geographically widespread incidence across the region. While mortality remains low in most countries for all four EVAs, spatial clustering of elevated mortality is evident in a limited number of settings, often coinciding with areas of high incidence. By contrast, the spatial distribution of case-fatality rates reveals distinct hotspots that are not always associated with the highest incidence areas. Notably, snakebites and spider bites account for the greatest number of countries with elevated case-fatality rates, concentrated primarily in tropical and subtropical regions for snakebites and in the Southern Cone for spider bites.

### Temporal trend in antivenom production and management

We identified a positive and statistically significant association between year and procurement for scorpion antivenoms (*β* = 0.1164; *P* = 0.0033) (Figure S1 and Table S5 in the **Online Supplementary Document**).

For *Lonomia* antivenoms, the therapeutic use exhibited a significant negative trend over time (*β* = −0.1531; *P* < 0.001). We did not identify any other statistically significant temporal trends.

### Estimating therapeutic preparedness relative to the epidemiological burden

Assessing the adequacy of the three components of the antivenom ecosystem in relation to the epidemiological burden for 2021–2024, we identified substantial discrepancies between estimated antivenom requirements and actual production, procurement, and use across all four envenomation categories (**Table 2**). For snakebites, both production (+35%) and procurement (+27%) exceeded the estimated need, whereas actual use was 35% below the estimated requirement. For scorpion stings, the estimated need greatly exceeded public-sector production, resulting in a 78% production deficit. Although procurement and use were closer to meeting expected needs, they remained insufficient (20% and 33% below, respectively). For spider bites, a severe and consistent deficit was noted across all components: production (−84%), procurement (−68%), and use (−76%). In cases of accidental contact with caterpillars, production (−3%) and procurement (−5%) were close to the estimated need; however, actual use was dramatically lower (−84%), pointing to substantial underutilisation.

**Table 2:** Comparison between the total number of antivenom vials produced, procured, or used and the estimated number of vials required to treat EVA cases in Latin America and the Caribbean (2021−2024)

	Number of produced vials (A)	Number of procured vials (B)	Number of used vials (C)	Number of EVA cases (D)	Average number of vials per treatment (E)*	Estimated number of vials used in EVA cases, D × E (F)	(A−F)/F, %	(B−F)/F, %	(C−F)/F, %
**Snake bite**	2,064,341	1,940,952	987,143	218,629	7	1,530,403	35	27	−35
**Scorpion sting**	432,204	1,568,452	1,314,125	1,959,824	1	1,959,824	−78	−20	−33
**Spider bite**	43,876	88,565	66,116	276,178	1	276,178	−84	−68	−76
**Caterpillar contact**	21,168	20,750	3,450	21,902	1	21,902	−3	−5	−84

EVA – envenomation by venomous animals

*We considered the average number of vials recommended for treatment by severity category (mild, moderate, and severe), as well as the average proportion of cases in each category, based on therapeutic guidelines [25–30] and previously reported data [8,31–43], respectively.

## DISCUSSION

### Epidemiological and programmatic challenges

Snakebites and scorpion stings remain the most important envenomations in LAC, accounting for the highest case-fatality rates and incidence, respectively. Nevertheless, our findings also highlight important changes in the epidemiology of other venomous animal accidents. The pronounced increase in caterpillar-related accidents observed in this study is consistent with the growing recognition of these events as an emerging regional public health threat [44]. This trend may also be associated with the recent identification of additional venomous *Lonomia* species causing accidents beyond the two classically implicated, *L. achelous* and *L. obliqua* [45], potentially contributing to an increase in human envenomation cases.

Though often recognised as an envenomation for which antivenom treatment is not frequently registered [3], spider-related accidents were the only EVA category showing a significant increase in deaths during the study period. This finding is particularly noteworthy because it occurred alongside a corresponding increase in reported cases and a projected increase in case-fatality rates. The observed pattern may be partly explained by improvements in mortality reporting in some countries, but it may also reflect persistent limitations in timely access to diagnosis and appropriate treatment.

The absence of statistically significant trends in deaths for the remaining categories should be interpreted cautiously. Given the relatively low frequency of fatal outcomes associated with these envenomations, the available time series may have limited statistical power to detect temporal changes despite regional aggregation. It is also possible that the number of deaths has remained relatively stable despite increasing numbers of reported cases, potentially reflecting improvements in clinical management, antivenom availability, and the overall therapeutic capacity of health systems in the region.

While temporal patterns provide insights into the evolution of EVAs over time, their spatial distribution reveals important differences in epidemiological burden across the region. Ecological, socio-environmental, and structural factors likely influence the occurrence and outcomes of these events in different ways across countries. Beyond national-level statistics, substantial heterogeneity in incidence and mortality may also occur across regions within the same country [6]. This indicates a need for differentiated strategies for surveillance, prevention, and healthcare organisation, guided by the specific epidemiological characteristics of each type of EVA in each territorial context.

Taken together, these findings highlight that the epidemiology of EVAs in LAC is dynamic and heterogeneous, with distinct temporal and spatial patterns across envenomation types. In this context, the projected increase in the case-fatality rate associated with spider-related accidents by 2030 is particularly concerning, as it underscores the urgency of strengthening early diagnosis, clinical management capacities, and access to effective antivenoms, while also improving ecological and epidemiological monitoring systems capable of detecting shifts in species distribution, venom toxicity, and patterns of human exposure. More broadly, these projected trends emphasise the importance of anticipating emerging risks and adapting health system responses to the evolving epidemiological landscape of EVAs in LAC

### The healthcare and surveillance context of EVAs in LAC

Across LAC, several countries have made substantial progress in establishing national programmes dedicated to the prevention, surveillance, and management of EVAs. However, some still lack systematic and protocolised programmes, resulting in high underreporting and variable clinical practices, uneven diagnostic capacity, and limitations in the implementation of standardised treatment protocols [6]. This denotes differences in resources, governance, and technical capacity among countries, remaining key barriers to achieving similar outcomes across the region (**Table 3**).

**Table 3:** Strengths and areas for improvement in healthcare and surveillance capacity for EVAs in Latin America and the Caribbean

Category	Strengths	Areas for improvement
**Surveillance and reporting systems**	Existence of mandatory notification for at least one type of EVA in most countries, providing a minimal epidemiological data foundation.	Significant underreporting, especially in remote areas with limited access to antivenom treatment and healthcare systems. Heterogeneous surveillance systems, lacking regional harmonisation. Minimal or no monitoring of physical and psychological sequelae, and lack of inclusion of disability indicators in national surveillance systems, particularly as a future priority after improvements in case notification.
**Diagnostic capacity and case management**	Availability of national guidelines/protocols for EVA management in primary care and hospital settings. Availability of training programmes to offer enhanced competencies for health professionals in EVA management. Availability of effective, safe and quality antivenoms from public manufacturing laboratories.	Most national clinical and therapeutics protocols require updating. Absence of an operational framework for permanent/continuous education in EVAs. Unequal access to healthcare and antivenoms in rural areas, leading to delayed treatment and increased complications.
**Mapping, risk assessment and early warning**	Existence of basic information on spatial patterns of EVAs in most countries. Regional awareness of the need to apply technology to map high-risk areas.	Weak mapping and risk-projection capacity, with minimal integration of climatic, urban, and ecological drivers. Absence of predictive systems to anticipate the expansion of venomous species influenced by climate change and/or urbanisation.

EVA – envenomation by venomous animals

Although most countries maintain mandatory notification for at least one category of EVA, there are still major gaps in reporting from remote and underserved areas, limited health system connectivity, and the absence of unified data standards across countries. By prioritising the strengthening of surveillance systems and ensuring that all EVAs are consistently designated as compulsory notifiable events, countries would generate more reliable, comparable, and timely data, thereby improving understanding of their actual magnitude, geographic distribution, and public health impact.

Other critical deficit concerns mapping, early warning and risk-projection capabilities, which remain poorly developed or inconsistently applied. Although recent studies emphasise the importance of using technology to identify high-risk zones [46–48], surveillance platforms across the region still lack systematic integration of environmental and climate-change drivers, despite evidence that climatic variability is facilitating the expansion of some venomous species and increasing human exposure [9,47]. As a result, national risk assessments are often reactive rather than predictive, limiting preparedness for emerging hotspots.

Despite the availability of diagnostic-therapeutic guidelines in most countries, they are not periodically updated, failing to incorporate evolving epidemiological patterns, such as the increasing incidence of scorpionism [7], or the most current scientific evidence on clinical management and surveillance [49]. Furthermore, the lack of structured and permanent training initiatives/programmes for health professionals limits the capacity of health teams to manage complicated or emerging cases, particularly in rural areas where these events most often occur. Thus, health systems continue to struggle with fragmented training models, heterogeneous clinical practices, and the lack of mechanisms ensuring rapid dissemination and incorporation of new evidence into national protocols.

Another major shortcoming involves the lack of systematic follow-up of physical, functional and psychological sequelae. This is almost exclusively limited to research settings [50–54], remaining largely undocumented in routine surveillance systems. There are thousands of survivors experiencing long-term impairments that affect productivity, quality of life, and socioeconomic stability [51,53–55]. The consequences can be even more devastating for children, who may be left with permanent disabilities that shape their entire life trajectory. Additionally, in remote and underserved regions, where specialised rehabilitation services are scarce or geographically inaccessible, these long-term impacts are further compounded by delayed or absent rehabilitative care, perpetuating cycles of disadvantage for affected families and communities [54].

### The One Health approach

One Health represents a robust paradigm for strengthening preparedness and response systems for these events since snakebites and other accidents by venomous animals occur at the interface of human, animal, and ecosystem health, being influenced by ecological disruption, socioeconomic vulnerability, and changing environmental conditions. These events not only represent a direct health burden among humans, but also precipitate profound financial repercussions, mainly related to loss of productivity due to long-term sequelae [51,53,55]. Moreover, their impact on domestic animals, primarily livestock, translates into tangible economic losses for rural households, which rely on these animals for sustenance and income [56]. Thus, due to the complex ecological, social, economic, and political contexts in which EVAs are embedded, an integrative One Health framework becomes necessary to adequately address their determinants and consequences [57].

However, as recently revealed [6], this approach is still underutilised to address EVAs in countries of the region. In fact, recent research stressed that the One Health framework must move beyond conceptual endorsement toward practical integration, involving coordinated surveillance, shared data systems, harmonised protocols, and co-designed interventions rooted in local ecological and sociocultural contexts [58]. But a major limitation to reach this is the lack of governance structures and enabling policies to support routine One Health collaboration among academic and non-academic actors, decision-makers, authorities, governing bodies, and a diverse set of society’s stakeholders.

An example of practical One Health operationalisation in the region is the multi-sectoral initiative developed in Honduras, which aligned the Ministry of Health of Honduras, PAHO/WHO, the National Autonomous University of Honduras, the Honduras Medical College, non-governmental organisations (*e.g.* Ixchel), and international partners (Instituto Clodomiro Picado from Costa Rica) to advance coordinated surveillance, clinical training, antivenom procurement and distribution, and public communication [59]. This interinstitutional effort demonstrates how political commitment, shared governance, and joint capacity-building can translate the One Health framework into concrete improvements in preparedness and case management, and thus offers a feasible template for other Latin American countries.

### The antivenom ecosystem in LAC

#### Temporal trend in antivenom production and management

The overall pattern observed across antivenom production, procurement, and utilisation indicators points to a relatively stable antivenom ecosystem at the regional level during the study period, despite important fluctuations among products and countries. This pattern may reflect a combination of relatively constant epidemiological demand, heterogeneous procurement practices among countries, and the limited duration of the observation period. It should also be noted that the analysis included antivenom procurement and use data from both public and private laboratories, but considered antivenom production only from public laboratories.

#### The antivenom production context

Major efforts have been made in antivenom production over the past decade, driven largely by public laboratories that play an essential role in ensuring access to these life-saving biologicals [60]. Regional assessments reveal a longstanding, yet fragile, manufacturing landscape currently composed of 12 public producers across eight countries of Central and South America. Some of these laboratories have made notable progress in modernising infrastructure, upgrading equipment, and optimising unit operations (from plasma fractionation to formulation and quality control), in alignment with Good Manufacturing Practices (GMP) [12,17]. However, despite these achievements, antivenoms continue to be considered ‘semi-orphan’ products [61], and some laboratories still face structural vulnerabilities, fluctuating production capacity, and discontinuities linked to infrastructure constraints, regulatory gaps, or supply-chain limitations. Marked heterogeneity among laboratories in the region calls for strengthening regional collaboration.

The establishment and consolidation of RELAPA represent one of the most transformative developments in recent years, providing an active platform for cooperation, technical training, knowledge exchange, research activities, and problem-solving among laboratories [11]. Activities, such as webinars, on-site training, peer-to-peer mentorship, and technical consultations, have been highly valued and have contributed to improvements in manufacturing processes, quality assurance systems, and workforce skills across the region [17]. Short- and medium-term objectives focus on supporting laboratories in achieving GMP compliance, standardising practices, and strengthening organisational capacity to ensure sustained, predictable, and high-quality production [12] to meet the growing demand for safe and effective antivenoms across LAC.

Interestingly, our findings revealed that reported production volumes of scorpion antivenoms were substantially lower than both procurement and utilisation levels. One possible explanation is that a significant proportion of these products may be supplied by private manufacturers whose production data were not captured in the regional data sets analysed. Nevertheless, the observed discrepancy highlights the importance of improving transparency and information-sharing on antivenom production across all sectors to support more accurate assessments of regional manufacturing capacity and supply sustainability.

Despite these advances, several limitations and challenges persist. First, it is important to highlight the lack of sustainable financial support from some governments, which would enable national laboratories to achieve and maintain GMP compliance. Such investment is urgently needed, despite the high costs, as it prevents countries from becoming dependent on commercial interests. This dependency is problematic not only because of supply fragility and high costs but also due to geographic variation in venom composition among medically important species across the region, which can affect neutralisation profiles and clinical antivenom efficacy. These factors underscore the need for resilient national-regional manufacturing strategies capable of preventing abrupt production gaps, safeguarding countries from prolonged reliance on external suppliers, and ensuring continuous access to context-appropriate, effective, and quality-assured antivenoms for the populations most at risk.

Second, communication between manufacturers and national regulatory authorities (NRAs) remains uneven. While some countries maintain strong, coordinated relationships that facilitate inspections, regulatory advice, and process improvements, others lack structured mechanisms for technical guidance, resulting in inconsistent oversight, absence of batch release procedures, or delays in achieving GMP certification [17]. As previously suggested [11], harmonised regulatory pathways and more frequent dialogue between manufacturing laboratories and NRAs are urgently needed. In this context, the PAHO Department of Innovation, Access to Medicines and Health Technologies (IMT) plays a key role by working with regional reference regulatory agencies across the Americas to advance regulatory harmonisation, promote information-sharing, and strengthen regional standards. This coordinated effort not only supports the development of more robust national quality-assurance systems but also helps laboratories overcome persistent barriers to international regulatory recognition, facilitates eligibility for procurement through multilateral mechanisms, and ultimately expands opportunities for the safe and reliable export of antivenoms throughout the region.

Third, the availability of human clinical studies validating antivenom efficacy and safety remains insufficient. Only a minority of laboratories report having clinical data, citing barriers such as limited financial resources, lack of teams, and infrastructure constraints for conducting clinical studies [17]. This gap compromises evidence-based deployment and undermines the ability to meet international regulatory expectations. Expanding multicentre clinical research, coupled with standardised methodologies, is therefore critical.

Fourth, post-marketing pharmacovigilance systems remain heterogeneous. Although some laboratories have established formal surveillance systems, many rely on partial or informal reporting, limiting the capacity to monitor adverse events and real-world effectiveness of antivenoms [17]. Systematic implementation of pharmacovigilance procedures, including Periodic Safety Update Reports (PSURs), electronic registries, and risk management plans, is essential to strengthen patient safety and regulatory compliance.

#### The antivenom procurement context

Planned antivenom procurement remains uneven across LAC, with several countries maintaining robust planning systems, established national programmes, and stable procurement mechanisms, frequently supported by national public manufacturers. In this study, we observed an increase in the procurement of scorpion antivenoms over time. This trend may reflect a growing recognition of scorpionism as an emerging public health concern in some areas of the region, consistent with recent evidence suggesting that medically important synanthropic scorpion species are expanding geographically into areas previously unaffected by scorpion stings [9]. In this context, procurement preparedness reflects strategic planning and readiness within the public health sector to ensure timely antivenom availability, reduce administrative bottlenecks, and optimise resource allocation. However, despite this encouraging trend, our study also revealed a probable scenario of incomplete therapeutic coverage and prolonged vulnerability in high-incidence settings, suggesting that recent increases in procurement may still be insufficient to meet the estimated therapeutic needs.

Although these observations are particularly relevant for scorpion antivenoms, they reflect broader challenges affecting antivenom procurement across the region. National programmes face structural challenges, including inconsistent epidemiological reporting, outdated treatment guidelines, insufficient stock monitoring, or even the absence of antivenoms for clinically relevant envenomations known to occur in the country. According to regional data [6], planning deficiencies have led many health systems to depend repeatedly on emergency donations, despite the complexity, cost, and long production timelines required for antivenoms.

A key aspect for improving procurement is the integration of rigorous preclinical efficacy data into national decision-making. Advances in toxinology, including detailed venom proteomics, toxicovenomics, and antivenomics, together with a set of animal-based laboratory methods to assess venom toxicity and neutralisation, now allow the preclinical determination of the neutralisation spectrum of antivenoms through *in vitro* and *in vivo* assays. Such tests are essential to define the geographical coverage and preclinical efficacy of each product, ensuring that procurement decisions are grounded in high-quality evidence rather than historical patterns or availability alone [14]. Significant efforts have been carried out in the region on the preclinical assessment of antivenom efficacy [62], and current projects are under way to further expand this body of information.

The procurement gaps identified in several countries further underscore the importance of regional cooperation mechanisms. The PAHO Strategic Fund [63] provides an organised platform for pooled procurement, quality assurance, and significant cost savings, and it is increasingly relevant as several countries depend on a limited number of public manufacturing laboratories, many of them integrated into RELAPA, for their antivenom supply. Leveraging such mechanisms, while embedding procurement within a framework informed by preclinical efficacy and neutralisation-spectrum data, is essential to move toward equitable, evidence based, and sustainable access to effective antivenoms throughout the region.

#### Antivenom availability and accessibility in settings of high incidence of EVAs

Ensuring timely patient access to health facilities equipped with effective, safe and high-quality antivenoms is critical to improving clinical outcomes and preventing complications, long-term sequelae, and death from EVAs [64]. However, cold-chain requirements, the need for adequately equipped health facilities, and the requirement for trained medical supervision generally restrict antivenom availability to hospitals, while primary healthcare settings are usually deprived of antivenoms [65]. At the regional level, antivenom access is predominantly concentrated in secondary and tertiary levels, ie, clinics and hospitals [6], most of which are located in urban areas. This concentration contributes to delays in antivenom administration for the most vulnerable sectors of the population, which inhabit rural, remote, and other high-risk areas, and these delays remain a major determinant of clinical outcomes, frequently leading to poorer prognoses [65].

Most concerning is that we identified here a persistent mismatch between therapeutic need and system capacity across all four EVA categories, with strong evidence that many cases requiring antivenom may not be receiving appropriate treatment, even in the case of snake antivenoms which showed a pattern of underutilisation despite adequate availability. This reveals structural and logistical shortcomings that demand urgent and coordinated regional action since such gaps have direct implications for timely case management, preventable morbidity and mortality, and health system resilience.

A similar situation was observed for *Lonomia* antivenoms. Despite the apparent availability of this therapeutic resource, utilisation remained considerably lower than expected based on the estimated treatment needs. This may reflect diagnostic challenges or clinical uncertainty, especially in non-endemic areas. However, given that we also observed a significant reduction in antivenom utilisation during the study period, the estimated therapeutic need may have been overestimated. Among several venomous caterpillar genera, only envenomation by *Lonomia* spp requires antivenom [44], which may partially explain the observed discrepancy. It is also possible that improvements in professional training and accumulated clinical experience have contributed to more accurate diagnoses, better case management, and consequently a more appropriate use of antivenom therapy.

An even more concerning situation was observed for spider antivenoms, for which we identified simultaneous deficits in production, procurement, and utilisation, indicating a critical weakness in therapeutic preparedness for this type of envenomation. Given the known morbimortality associated with *Latrodectus*, *Loxosceles* and *Phoneutria* envenomation [66], this gap raises serious concerns regarding preventable complications.

Addressing these gaps will require not only strengthening antivenom supply chains but also improving the accessibility of available products. Several countries in the region have begun to address this limitation by implementing innovative strategies to expand access at the primary healthcare level. Examples include the SAVING Program in Brazil, which promotes the decentralisation of snake antivenom treatment to indigenous health units in the Brazilian Amazon [67], and, more recently, the initiative by the Ministry of Health of Costa Rica authorising paramedic personnel to apply antivenoms in extra-hospital settings when patients face significant barriers to reaching formal health facilities [14]. Although the outcomes of these initiatives have not yet undergone full formal evaluation, preliminary evidence from the former demonstrates substantial improvements in timely access to antivenom, reductions in severe cases, mortality, and sequelae, and strengthened local clinical capacity in remote health units [68]. Building on these encouraging findings, similar public health policies that expand the availability and administration of antivenoms to indigenous communities and other rural or hard-to-reach territories should be actively supported, scaled, and adapted across all countries in the region to advance equity and reduce preventable treatment delays.

### Limitations

Overall, our findings should be interpreted in the light of various limitations. First, epidemiological findings rely primarily on data reported by countries, which are often subject to underreporting and may be strongly influenced by national public policies and strategic plans. Moreover, substantial heterogeneity in case notification rates across countries may affect the accuracy of the regional models. Additionally, stochastic variation may be associated with the short observation period and the relatively small number of reported events for some EVA categories.

Second, although procurement and use data included both public and private sources, production data were restricted to public laboratories, potentially underestimating regional manufacturing capacity for some antivenom categories.

Third, the number of vials required per patient differs across antivenoms, depending on their specificity, potency, manufacturer recommendations, and/or national therapeutic guidelines. Although we estimated the average number of vials per treatment based on multiple national and institutional guidelines, differences in recommended dosing schemes and severity classifications across jurisdictions may affect the comparability and validity of these estimates. Finally, substantial heterogeneity in procurement processes, storage capacity, case management protocols, and reporting practices across countries limits the extent to which these indicators can be compared and extrapolated at the regional level.

Notwithstanding these limitations, the study identifies several consistent epidemiological and programmatic patterns that have direct implications for public health decision-making. The findings reveal important challenges affecting both the prevention and management of EVAs and the sustainability of the antivenom ecosystem, providing a robust evidence base to inform the strategic directions outlined in the following recommendations.

### Recommendations for future policies

Aligning with the WHO global strategy’s objective of mitigating the multifaceted repercussions of envenomation [5], countries across LAC should adopt a shift from fragmented, reactive approaches toward coordinated, forward-looking strategies. The coming years present an opportunity to move beyond emergency-driven responses and instead consolidate a regional ecosystem capable of preventing, detecting and managing these events, ensuring timely access to safe, effective, and context-appropriate antivenoms. The following subsections outline the three interconnected priority areas that could be critical to shaping a resilient and more equitable future for prevention and control of EVAs across the region.

#### Strengthening national programmes and health systems

Integrating EVAs within broader neglected tropical disease and public health agendas within a One Health paradigm could not only increase its visibility as a regional priority but also enable countries to benefit from existing infrastructures and local resources to create a robust and sustainable health system by means of the following priority actions.

### Standardising and expanding compulsory reporting

Expanding mandatory notification to include all major EVA types is essential to allow a better understanding of their epidemiology, particularly as climate change and environmental alterations accelerate shifts in species distribution. Standardised, timely, and interoperable reporting should also be positioned as a core public health function to ensure that envenomation surveillance keeps pace with evolving ecological and epidemiological realities. In this regard, the Brazilian Notifiable Diseases Information System is a good example of a consistent compulsory reporting system available that has been progressively improving the accuracy of incidence, severity, and mortality estimates, and thus has enabled better identification of regional risk patterns, and detection of temporal trends [69].

Additionally, a harmonised regional reporting framework would enable countries to generate comparable data and strengthen collective situational awareness, allowing for more precise identification of cross-border patterns and emerging threats. Regional standardisation would also support joint forecasting of antivenom needs, improve stock management through coordinated procurement and distribution strategies, and facilitate targeted prevention and risk-communication efforts adapted to ecological, demographic, and occupational vulnerabilities. Establishing such a framework would not only enhance the quality and usability of national surveillance data but also serve as a foundation for regional cooperation mechanisms capable of responding more rapidly and effectively to the evolving burden of EVAs across LAC.

### Risk mapping and integration of climate-sensitive risk modelling

Having risk maps, not only at national level but also at district and municipal level, is an urgent cornerstone for optimising resource allocation and targeting life-saving interventions. But this can pose a challenge when based solely on officially reported cases since they are usually concentrated near cities or urban areas (where hospital density is higher), hindering hotspot detection [70,71]. New digital tools, including artificial intelligence-enabled mobile applications such as SERPENT-Brasil [72], could help in this regard. By enabling users to register cases and snake sightings, this application generates real-time data that may inform public health planning and help identify high-risk areas even in remote settings [72]. Another official but not specific application for venomous animals is the wildlife health information system, SISS-Geo, developed through a partnership between the Brazilian Ministry of Health and the Oswaldo Cruz Foundation. As a citizen–science tool, it allows citizens, health and environmental professionals, researchers and specialists to contribute with georeferenced observations of animals, enabling surveillance and prevention actions for health.

Critically, global environmental change and anthropic activities will shift EVAs risk through interacting climate and land-use mechanisms: warming and altered rainfall can expand or contract suitable ranges and population densities of medically relevant species, while deforestation, agricultural expansion and urbanisation can reorganise local assemblages, sometimes increase encounters or create new corridors for invasive venomous species [73]. Integrating these drivers into risk maps is essential to anticipate emerging hotspots and pre-position antivenoms accordingly.

### Improving healthcare delivery and outcomes

Strengthening the clinical management of EVAs in LAC requires a comprehensive approach centred on updated evidence-based care, skilled health professionals, and the incorporation of agile, accessible, and context-relevant technological tools. Updated clinical protocols and therapeutic guidelines are fundamental to improving outcomes, reducing complications, and harmonising practices across diverse health systems. In this regard, initiatives such as the recently released manual developed by PANAFTOSA/PAHO [49], offer an essential reference for health professionals, providing region-specific guidance on diagnosis, risk stratification, antivenom use, and supportive care for snakebites. The manual is complemented by an online course [74] designed to expand knowledge among frontline healthcare workers and reinforce the adoption of these harmonised practices across countries in the region. Similar activities are being planned for the near future with respect to other EVAs.

Despite these regional efforts, persistent gaps in knowledge among healthcare workers of both urban referral centres and remote rural settings [72] highlight the need for ongoing capacity-building efforts that incorporate updated scientific evidence, case management algorithms, and context-appropriate decision tools, such as the clinical protocols and therapeutic guidelines for snakebites and scorpion envenomations that recently came into official effect in Brazil [26,27]. Emphasis should also be placed on integrating these topics into medical and nursing school/university curricula. Furthermore, the expansion of digital health tools represents a strategic opportunity to enhance access to information and education. A notable example in this regard is the Venomous Animals app, which is an initiative of the Brazilian Ministry of Health offering a complete platform to support both the public and health professionals during snakebite emergencies, improving snakebite management and prevention, easing the burden on health services, and promoting health education.

### Integrating systematic reporting of sequelae and long-term follow-up

National surveillance systems should structure an integrated reporting of physical, psychological, and socio-functional sequelae resulting from EVAs. This should include standardised indicators and interoperable data platforms that enable longitudinal monitoring of affected individuals. Building on these enhanced surveillance mechanisms, countries should establish integrated programmes that provide coordinated physical rehabilitation, mental health support, and social reintegration services. Such programmes are essential to identify, monitor, and accompany patients experiencing long-term complications.

Moreover, embedding long-term follow-up pathways within primary healthcare networks can help ensure continuity of care, reduce preventable disability, and generate robust evidence to guide policies, optimise antivenom distribution, and refine clinical guidelines. This comprehensive approach recognises that the burden of EVAs extends far beyond the acute episode and that sustained, multidisciplinary care is fundamental to achieving equitable health outcomes in the region.

#### Ensuring equitable and timely access to safe, effective, and quality antivenoms

Achieving this goal requires strengthening the entire antivenom ecosystem, from production and regulatory oversight to forecasting, procurement, and distribution to ensure that life-saving treatments reach the populations most at risk. The following priority areas outline key strategies to advance these objectives and reinforce the resilience of the antivenom ecosystem throughout the region.

### Enhancing antivenom production and regulatory harmonisation

Implementing a coordinated regional strategy, building on the platform and technical cooperation capacities of RELAPA, is essential to advance regulatory harmonisation and strengthen the quality, safety, and effectiveness of antivenoms. Priority actions include ensuring that all public and private manufacturers obtain and maintain GMP certification aligned with international standards; developing and adopting standardised protocols to generate clinical evidence on antivenom effectiveness; and institutionalising the routine submission of PSURs and comprehensive risk-management plans. Equally critical is the reinforcement of pharmacovigilance and post-market surveillance systems through clear regulatory mandates, interoperable reporting platforms, and systematic inclusion of antivenom-related adverse events in national and regional monitoring schemes. Together, these measures would help reduce heterogeneity in product quality, support regulatory convergence, and strengthen confidence among healthcare workers, regulatory authorities, and manufacturers, contributing to a safer and more transparent antivenom ecosystem.

### Strengthening strategic planning

Countries should prioritise systematic and forward-looking antivenom planning grounded in robust epidemiological intelligence, integrating geospatial modelling, climate-sensitive risk mapping, and real-time surveillance for early detection of geographic shifts in incidence. Strategic forecasting should replace the persistent regional dependence on emergency donations and reactive procurement cycles, enabling health authorities to anticipate annual and seasonal demand with greater precision, improve resource allocation, and establish more resilient antivenom supply chains. Incorporating local epidemiological determinants, such as agricultural activities, road accessibility, and indigenous population distribution, can further refine estimates of need and support context-specific preparedness strategies.

### Decentralising antivenom availability

To ensure that patients receive prompt and appropriate care, countries should promote the decentralisation of antivenom distribution, prioritising high-incidence, remote, and underserved areas, particularly within primary healthcare networks. Strengthening distribution logistics, cold-chain capacity, and rapid-replenishment mechanisms is vital to avoid stockouts and minimise delays in treatment initiation. Decentralised strategies should be supported by continuous training of frontline healthcare workers in diagnosis, case management, and rational use of antivenoms to ensure that expanded access is matched by clinical readiness.

#### Promoting operationalisation of One Health

Some countries of the region have successfully addressed some other health threats through intersectoral coordination, linking public health, veterinary services, environmental management, agriculture, and community governance [75], and these platforms could serve as effective models for EVAs. Such a mechanism would enable joint surveillance, harmonised risk assessment, and coordinated response actions, for example by linking epidemiological reporting of snakebites and scorpion stings with environmental monitoring of species distribution and local ecological drivers, and by leveraging community engagement to detect and prevent incidents early. Additionally, those platforms can support joint planning and shared logistics (*e.g.* coordinated procurement and distribution of antivenom and vaccine supplies to rural facilities, and joint training for frontline healthcare workers).

Because governance and legal frameworks are central to sustaining cross-sectoral action, countries should institutionalise One Health practices through legislation, inter-ministerial agreements, and anchored budget lines. By aligning mandates, budgets, data systems, and workforce competencies across ministries and agencies, countries can convert fragmented efforts into an integrated risk-management platform capable of anticipating eco-epidemiological shifts, implementing timely interventions, and thus preventing and reducing morbidity and mortality from EVAs in LAC.

## CONCLUSIONS

EVA cases continue to represent an expanding public health challenge in LAC, driven by environmental change, shifting distributions of medically important species, and persistent inequities in health system capacity. Although mortality remains relatively low for most EVAs, the projected increase in case-fatality rate associated with spider envenomation and the emergence of *Lonomia* accidents point to potentially urgent threats. The findings show that national surveillance systems remain fragmented, underreporting is widespread, and clinical protocols are not consistently updated or implemented across countries. Moreover, long-term physical, psychological, and socioeconomic sequelae remain largely unrecognised within routine health system monitoring, perpetuating the chronic and often invisible burden associated with these neglected conditions.

The antivenom ecosystem remains fragile. Production by public laboratories has improved but remains heterogeneous and vulnerable to financial, regulatory, and logistical constraints. Procurement processes in several countries continue to rely on emergency responses rather than strategic planning, and therapeutic use frequently diverges from epidemiological need, leaving many high-risk populations without timely access to effective antivenoms.

In interpreting these findings, some limitations should be considered. The data were derived from policy-driven, multi-country surveys, which may introduce variability in data quality and potential reporting biases. Additional constraints include underreporting, heterogeneity across data sources, and limited availability of systematic information on outcomes and follow-up. These factors may influence the precision and comparability of the results across settings.

Notwithstanding these limitations, this study provides a comprehensive regional assessment based on official data from ministries of health and public antivenom manufacturers, offering a structured and comparative perspective that has not been previously presented in a systematic manner. The findings highlight critical gaps that require coordinated, evidence-informed regional action. Key priorities include strengthening compulsory, harmonised reporting; integrating climate-sensitive risk mapping; updating clinical guidelines; and expanding training for frontline health providers are essential steps. Equally important are improving antivenom distribution, forecasting, and regulatory harmonisation to ensure quality, safety, and continuity of supply. Advancing One Health approaches can further enhance multisectoral coordination, linking environmental monitoring, health surveillance, and community engagement.

Overall, LAC stands at a pivotal moment to transition from reactive responses to proactive, integrated strategies. By consolidating recent regional initiatives and strengthening health system capacities, countries can significantly reduce the preventable burden of envenomation, advance equity, and accelerate progress toward the WHO 2030 goals.

## Additional Material


Online Supplementary Document

## Data Availability

**Data availability:** All data relevant to the study are included in the article or its supplementary document. Any other data can be obtained upon reasonable request from the corresponding authors.
