# Supplementary material for: Strengthening public health programmes and the antivenom ecosystem: strategic policy priorities to combat envenomation by animal bites and stings in Latin America and the Caribbean
Source: J Glob Health. 2026 Oct 9;16:04329. doi: 10.7189/jogh.16.04329 (PMC13650973; doi:10.7189/jogh.16.04329)
Supplement: Supplementary file 1 [file jogh-16-04329-s001.pdf]

Supplement to: Peichoto M, Gutiérrez J, Fan H, Damasceno C, Vigilato M; RELAPA, REDPEVA. Strengthening public health programmes and the antivenom ecosystem: strategic policy priorities to combat envenomation by animal bites and stings in Latin America and the Caribbean. J Glob Health. 2026;16:04239.

Table S1. Adherence to JoGH's GRABDROP guidelines items.

**JoGH guideline items**
1. Please list all papers published by each co-author in previous 3 years that were based on secondary analysis of a big data repository
- Favalesso MM, Valentim M, Guimarães ATB, Peichoto ME. Epidemiological characterization of lonomism in South America. Acta Trop. 2023;238:106777. https://doi.org/10.1016/j.actatropica.2022.106777.<br>- Favalesso MM, Valentim M, Martínez ME, de Oliveira Junior AC, Lorini LM, Martins Silva S, Guimarães ATB, Peichoto ME. Unraveling an overlooked risk factor for envenoming: Relationship between Lonomia caterpillars and their host plants. Oecologia Australis 2026. https://revistas.ufrj.br/index.php/oa/article/view/67650.<br>- Martínez PA, Moura TA, Barrios-Montivero AE, Gutiérrez JM, Boschemeier AGE. Deep learning forecasting of scorpion envenoming incidence in Brazil to support early warning and prevention: temporal validation across 27 states. Communications Health, 2026; 1: 2. https://doi.org/10.1038/s44528-026-00002-9.<br>- Moura TA, Gutiérrez JM, Silva FF, Sasa M, Siquiera-Silva T, Martínez PA. AI models uncover factors influencing scorpionism in Northern Brazil. Toxicon, 258: 108342. https://doi.org/10.1016/j.toxicon.2025.108342.<br>- Isaacson JE, Ye JJ, Silva LL, Hernandes Rocha TA, de Andrade L, Scheidt JFHC, Wen FH, Sachett J, Monteiro WM, Staton CA, Vissoci JRN, Gerardo CJ. Antivenom access impacts severity of Brazilian snakebite envenoming: A geographic information system analysis. PLoS Negl Trop Dis 2023; 17(6): e0011305. https://doi.org/10.1371/journal.pntd.0011305<br>- Zimmerman A, Monteiro WM, Vissoci JRN, Smith ER, Rocha T, Sachett J, Wen FH, Staton C, Gerardo CJ, Ogbuoji O. Scaling up antivenom for snakebite envenoming in the Brazilian Amazon: a cost-effectiveness analysis. The Lancet Regional Health – Americas 2024;29:100651. https://doi.org/10.1016/j.lana.2023.10065.1pntd.0011305.<br>- Sachett A, Strand E, Serrão-Pinto, Silva Neto A, Nascimento TP, Jati SR, Rocha GS, Andrade AS, Wen FH, Pucca MB, Vissoci J, Gerardo CJ, Sachett J, Farias AS, Monteiro WM. Capacity of Community health centers to treat snakebite envenoming in indigenous territories of the Brazilian Amazon. Toxicon 2024;241 April 2024, 107681.<br>- Rocha TAH, Silva LL, Wen FH, Sachett J, Tupetz A, Staton CA, Monteiro, Vissoci JRN, Gerardo CJ. River dataset as a potential fluvial Transportation network for healthcare access in the Amazon region. Scientific data 2023;10:188. https://doi.org/10.1038/s41597-023-02085-3.<br>- Lacerda AB, Candido D, Azevedo TS, Wen FH, Freitas GD, Dourado FS, Chiaravalloti Neto F. Scorpionism in Brazil: Space-time approach and risk areas in 2012 to 2024. PLoS Negl Trop Dis 19(10): e0013602. https://doi.org/10.1371/journal.pntd.0013602.<br>- Chiaravalloti-Neto F, Lorenz C, Lacerda AB, Azevedo TS, Candido DM, Eloy LJ, Wen FH, Blangiardo M, Pirani M. Spatiotemporal bayesian modelling of scorpionism and its risk factors in the state of São Paulo, Brazil. PLoS Negl Trop Dis 17(6): e0011435. https://doi.org/10.1371/journal.pntd.0011435.<br>- Janko MM, Araujo AL, Ascencio EJ, Guedes GR, Vasco LE, Santos RO, Damasceno CP, Medrano PG, Chacón-Uscamaita PR, Gunderson AK, O'Malley S, Kansara PH, Narvaez MB, Coombes C, Pizzitutti F, Salmon-Mulanovich G, Zaitchik BF, Mena CF, Lescano AG, Barbieri AF, Pan WK. Study protocol: improving response to malaria in the Amazon through identification of inter-community networks and human mobility in border regions of Ecuador, Peru and Brazil. BMJ Open. 2024 Apr 15;14(4):e078911. https://doi.org/10.1136/bmjopen-2023-078911.<br>- Molina-Flores, B.; Vigilato, M.A.N.; Rocha, F.; Cossivi, O.; Corrales, M.; Vásquez Niño, G.A.; Faccini-Martínez, Á.A.; Chiba de Castro, W.A.; Biondo, A.W.; Cediel-Becerra, N. Assessment of One Health Initiatives from a Veterinary Public Health Approach in Latin America and the Caribbean. Trop. Med. Infect. Dis. 2025, 10, 315. https://doi.org/10.3390/tropicalmed10110315.

- Boutelle, C., Mollentze, N., Gigante, C., Rocha,F., Vigilato, M.A.N., *et al.*. A logistic regression model to predict the next rabies virus host-shift event. *Sci Rep* **15**, 19306 (2025). https://doi.org/10.1038/s41598-025-98986-x.
- Rocha, F., Sibim, A. C., Molina-Flores, B., Chiba de Castro, W. A., Kmetiuk, L. B., Alves, R. V., dos Santos, A. L. d. S., Moreno, M. C., Faccini-Martínez, Á. A., Cediel, N. M., Biondo, A. W., Cosivi, O., & Vigilato, M. A. N. (2024). One Health Priorities: Advancing Veterinary Public Health in Latin America and the Caribbean. Pathogens, 13(8), 710. https://doi.org/10.3390/pathogens13080710.
- Saboyá-Díaz MI, Maia-Elkhoury ANS, Luciañez A, Valadas SYOB, Carvaho-Scholte RG, Nicholls RS, Vigilato MAN, Castellanos LG and Espinal MA (2024) Neglected infectious diseases in the Americas: current situation and perspectives for the control and elimination by 2030. Front. Trop. Dis 5:1326512. https://doi.org/10.3389/fitd.2024.1326512.

2. Please explain the key elements of your study design and the use of the available datasets that make your study an original scientific contribution

The study provides insights that go beyond descriptive reporting and constitutes an original scientific contribution to the field by generating a novel, system-level analytical framework that quantifies and models the relationship between epidemiological burden, health system capacity, and antivenom ecosystem performance in LAC. Importantly, this approach translates heterogeneous and policy-oriented data into robust, comparable scientific evidence, thereby bridging the gap between research and practice and offering actionable information to support evidence-informed decision-making and strategic policy development at the regional level.

3. Please list all publications that addressed similar research questions in the same dataset and indicate where you cited them in your paper

To the best of our knowledge, no previous publications have addressed similar research questions using the same datasets (i.e., the combined REDPEVA and RELAPA survey data analyzed in this study). While some regional reports (cited throughout the manuscript) have summarized portions of these data, they have not applied the integrated analytical framework, quantitative modeling, and cross-component (epidemiology-antivenom ecosystem) assessment presented here.

4. Please explain how you addressed multiple testing through an appropriately rigorous statistical threshold and indicate this in the methods section

In the present study, the regression analyses were not intended to serve as a broad exploratory screening of independent associations, but rather as a set of pre-specified temporal trend analyses, applied consistently to a limited number of epidemiologically defined outcomes: cases and deaths for the four main types of envenomation, and indicators of production, procurement, and use for the corresponding categories of antivenom.

For this reason, we did not apply a formal post hoc correction for multiple comparisons, as it would be excessively conservative given the small number of observations, the correlation among outcomes, and the descriptive public health objective of the analysis. Instead, we used a conventional statistical threshold of $p < 0.05$, interpreted in conjunction with effect size, direction of association, 95% confidence intervals, epidemiological plausibility, and the consistency of results across related indicators. We also avoided interpreting isolated p-values as definitive evidence and explicitly framed projections as illustrative rather than predictive. Thus, the statistical results were used to support the identification of regional temporal patterns and health management priorities, rather than to establish confirmatory causal inference.

5. Please declare to what extent have AI chatbots been used in developing your paper and to which parts of the paper did they contribute

During the preparation of this manuscript, the authors used ChatGPT in order to adjust the different R scripts, and DeepL or Microsoft Copilot in order to support text revision and language editing. After using these tools, the authors thoroughly reviewed and edited the content as needed and take full responsibility for the content of the manuscript.

Table S2. Details on data source for countries in this study.

Country	Source	Years	Data status	Neighbor(s) used	Animal involved in events
Argentina	REDPEVA	2019-2024	Observed	–	Snake, Scorpion, Spider, Caterpillar
Belice	REDPEVA	2019-2024	Observed	–	Snake, Scorpion, Spider, Caterpillar
Bolívia	REDPEVA	2021-2024	Observed	–	Snake, Scorpion, Spider
Brazil	REDPEVA	2019-2024	Observed	–	Snake, Scorpion, Spider, Caterpillar
Chile	REDPEVA	2019-2020	Observed	–	Snake, Scorpion, Spider, Caterpillar
Colombia	REDPEVA	2019-2024	Observed	–	Snake, Scorpion (2019, 2020 and 2024), Spider (2019, 2020 and 2024), Caterpillar (2019, 2020 and 2024)
Costa Rica	REDPEVA	2019-2024	Observed	–	Snake, Scorpion, Spider (2019-2020), Caterpillar
Cuba	REDPEVA	2021-2024	Observed	–	Snake, Scorpion, Spider, Caterpillar
Ecuador	REDPEVA	2019-2024	Observed	–	Snake, Scorpion, Spider, Caterpillar
El Salvador	REDPEVA	2021-2024	Observed	–	Snake, Scorpion, Spider, Caterpillar
Guatemala	REDPEVA	2019-2024	Observed	–	Snake, Scorpion, Spider, Caterpillar
Guayana	Literature*	2024	Observed	–	Snake
Guayana Francesa	REDPEVA	2021-2024	Observed	–	Snake, Scorpion
Haiti	REDPEVA	2021-2024	Observed	–	Snake, Scorpion, Spider, Caterpillar
Honduras	REDPEVA	2019-2024	Observed	–	Snake, Scorpion, Spider, Caterpillar (2019-2020)
Jamaica	REDPEVA	2021-2024	Observed	–	Snake
México	REDPEVA	2019-2024	Observed	–	Snake (2021-2024), Scorpion, Spider, Caterpillar (2019-2020)
Nicaragua	REDPEVA	2019-2024	Observed	–	Snake, Scorpion, Spider, Caterpillar (2019-2020)
Panama	REDPEVA	2021-2024	Observed	–	Snake, Scorpion
Paraguai	REDPEVA	2019-2024	Observed	–	Snake, Scorpion, Spider, Caterpillar (2019-2020)
Peru	REDPEVA	2019-2024	Observed	–	Snake, Scorpion, Spider, Caterpillar
Saint Lucia	REDPEVA	2021-2024	Observed	–	Snake
Suriname	none	none	Imputed	Guayana & Guayana Francesa	Snake, Scorpion
Uruguay	REDPEVA	2019-2024	Observed	–	Snake, Scorpion (2019-2020), Spider, Caterpillar
Venezuela	REDPEVA	2019-2020	Observed	–	Snake, Scorpion, Spider, Caterpillar

* Song B, Rickett M, Hoyte C, Mughal A. A Review of Snakebite Envenoming at a Tertiary Referral Hospital Emergency Department in Guyana. Annals of Emergency Medicine. 2025;86:24–5.

Table S3. Estimates of the negative binomial model coefficients for the temporal trend of the number of cases by type of EVA (2019-2024). This presents the coefficients for the intercept and year terms obtained from negative binomial models adjusted separately for each type of EVA, considering the annual number of cases across countries as the result. The coefficient associated with the year represents the log-linear temporal trend in the number of cases, adjusted for population mean (offset). Positive values indicate an increasing trend, and negative values indicate a decreasing trend over the period.

Cases of	Term	Coefficient (β)	Standard error	Wald statistic	p-value
Snake	Intercept	-9.487	0.032	-298.188	<0.0001
Bite	Year	0.032	0.011	3.069	0.0021
Scorpion	Intercept	-7.275	0.042	-172.272	<0.0001
Sting	Year	0.032	0.014	2.292	0.0219
Spider	Intercept	-9.690	0.148	-65.460	<0.0001
Bite	Year	0.147	0.049	2.970	0.0030
Caterpillar	Intercept	-13.267	0.365	-36.344	<0.0001
Contact	Year	0.433	0.126	3.443	0.0006

The statistical significance of the intercepts indicates that the value of the dependent variable (Y) was significantly different from zero when the independent variable (X) was zero, which was expected and does not require additional interpretation.

Table S4. Estimates of the negative binomial model coefficients for the temporal trend of the number of deaths by type of EVA (2019-2024). This presents the coefficients for the intercept and year terms obtained from negative binomial models adjusted separately for each type of EVA, considering the annual number of deaths across countries as the result. The coefficient associated with the year represents the log-linear temporal trend in the number of deaths, adjusted for population mean (offset). Positive values indicate an increasing trend, and negative values indicate a decreasing trend over the period.

Deaths by	Term	Coefficient (β)	Standard error	Wald statistic	p-value
Snake bite	Intercept	-15.009	0.124	-120.967	<0.0001
	Year	0.032	0.041	0.788	0.4304
Scorpion sting	Intercept	-15.307	0.153	-100.299	<0.0001
	Year	-0.002	0.051	-0.035	0.9720
Spider bite	Intercept	-16.743	0.114	-146.681	<0.0001
	Year	0.179	0.033	5.421	<0.0001
Caterpillar contact	Intercept	-20.458	0.736	-27.798	<0.0001
	Year	0.246	0.209	1.175	0.2400

The statistical significance of the intercepts indicates that the value of the dependent variable (Y) was significantly different from zero when the independent variable (X) was zero, which was expected and does not require additional interpretation.

Table S5. Estimates of the negative binomial model coefficients for the three core dimensions of the antivenom ecosystem: production, procurement and use of antivenoms in LAC (2021-2024). This presents the coefficients for the intercept and year terms for the models fitted separately by antivenom type. The models assess the temporal trend in the number of produced/procured/used vials. The coefficient associated with the year represents the log-linear temporal variation in the number of vials.

**Antivenoms against**		**Term**	**Coefficient (β)**	**Standard error**	**Wald statistic**	**p-value**
Snake Bite	Production	Intercept	-118.847	403.659	-0.294	0.7684
		Year	0.062	0.200	0.312	0.7547
	Procurement	Intercept	22.502	256.495	0.088	0.9301
		Year	-0.007	0.127	-0.053	0.9577
	Use	Intercept	-132.771	187.360	-0.709	0.4785
		Year	0.069	0.093	0.749	0.4540
Scorpion Sting	Production	Intercept	-428.814	904.513	-0.474	0.6354
		Year	0.214	0.448	0.478	0.6327
	Procurement	Intercept	-229.096	80.193	-2.857	0.0043
		Year	0.116	0.040	2.937	0.0033
	Use	Intercept	-317.187	374.985	-0.846	0.3976
		Year	0.159	0.185	0.859	0.3902
Spider Bite	Production	Intercept	0.002	852.592	0.000	1.0000
		Year	0.002	0.422	0.005	0.9956
	Procurement	Intercept	69.771	180.520	0.386	0.6991
		Year	-0.032	0.089	-0.354	0.7232
	Use	Intercept	-319.846	1218.987	-0.262	0.7930
		Year	0.161	0.603	0.267	0.7897
Lonomia Contact	Production*	Intercept	0.000	-	-	-
		Year	0.004	-	-	-
	Procurement	Intercept	-65.962	453.229	-0.146	0.8843
		Year	0.036	0.224	0.160	0.8732
	Use	Intercept	314.605	74.976	4.196	<0.0001
		Year	-0.153	0.037	-4.132	< 0.0001

*For this case, the production model did not converge adequately, resulting in undefined estimates, possibly reflecting a low count and/or an excess of zeros in the data.

A

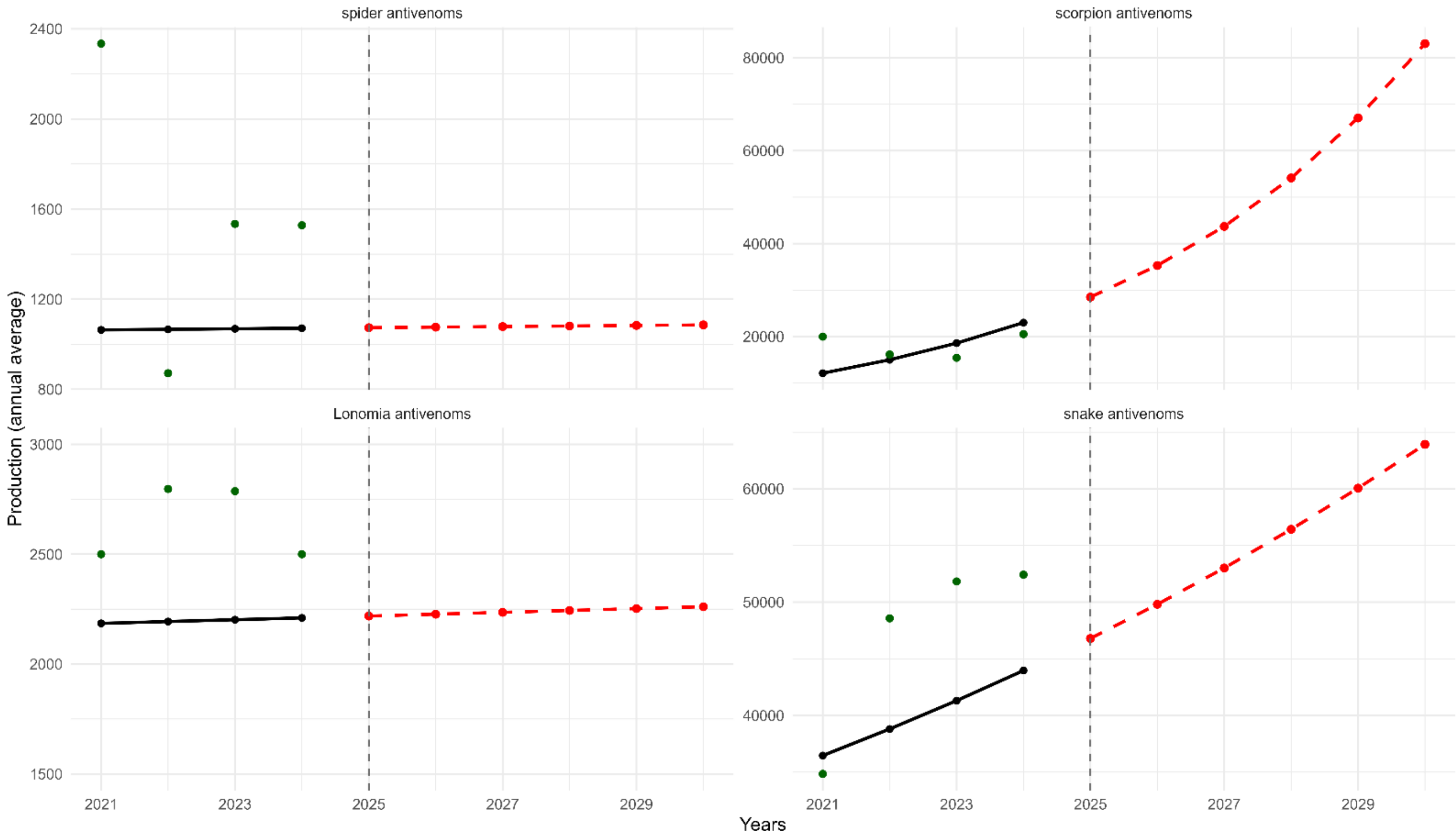
spider antivenoms
scorpion antivenoms
Lonomia antivenoms
snake antivenoms
Production (annual average)
Years
2400
2000
1600
1200
800
80000
60000
40000
20000
3000
2500
2000
1500
60000
50000
40000
2021
2023
2025
2027
2029

B

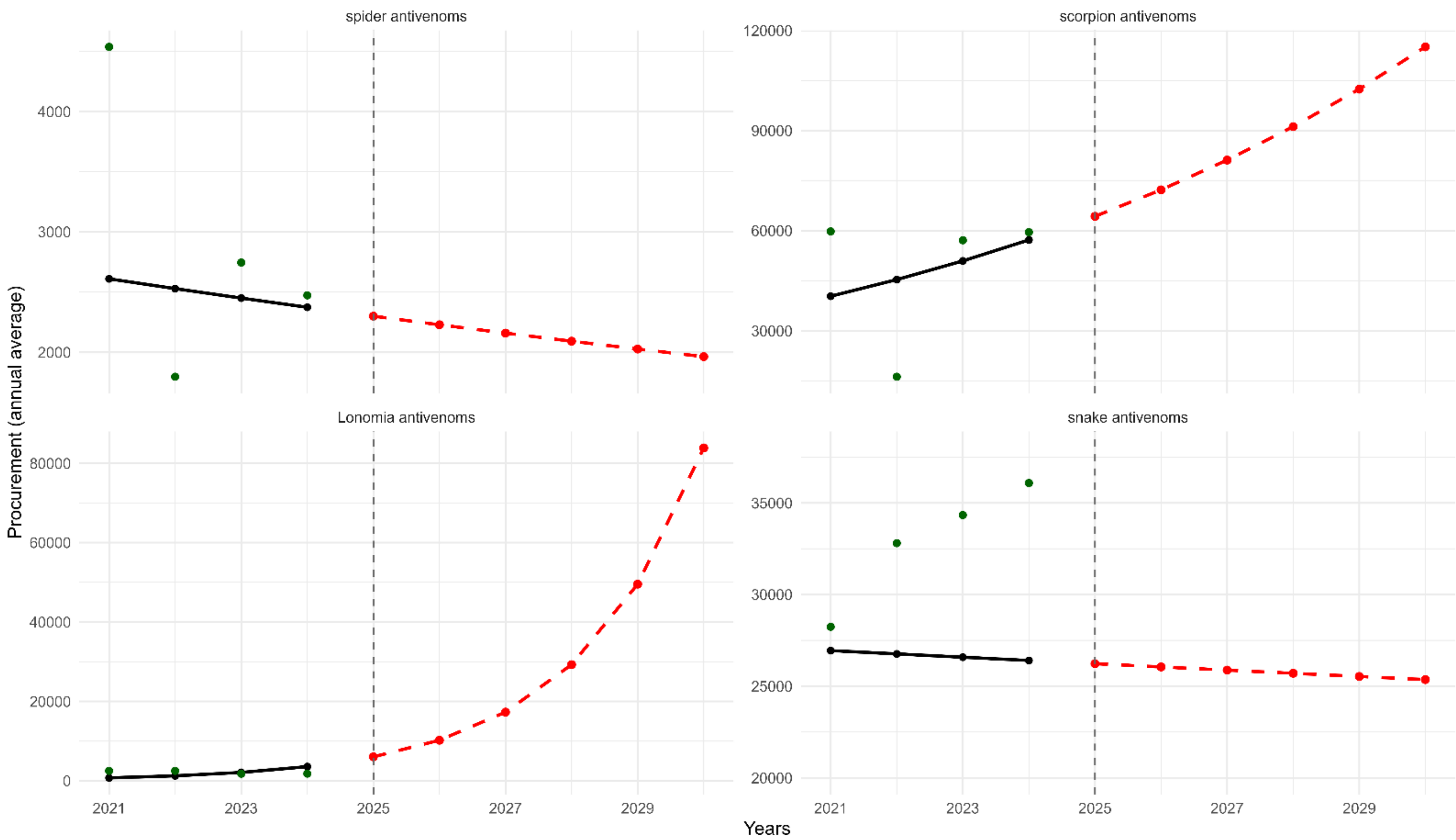
spider antivenoms
scorpion antivenoms
Lonomia antivenoms
snake antivenoms
Procurement (annual average)
Years
4000
3000
2000
120000
90000
60000
30000
80000
60000
40000
20000
0
35000
30000
25000
20000
2021
2023
2025
2027
2029

C

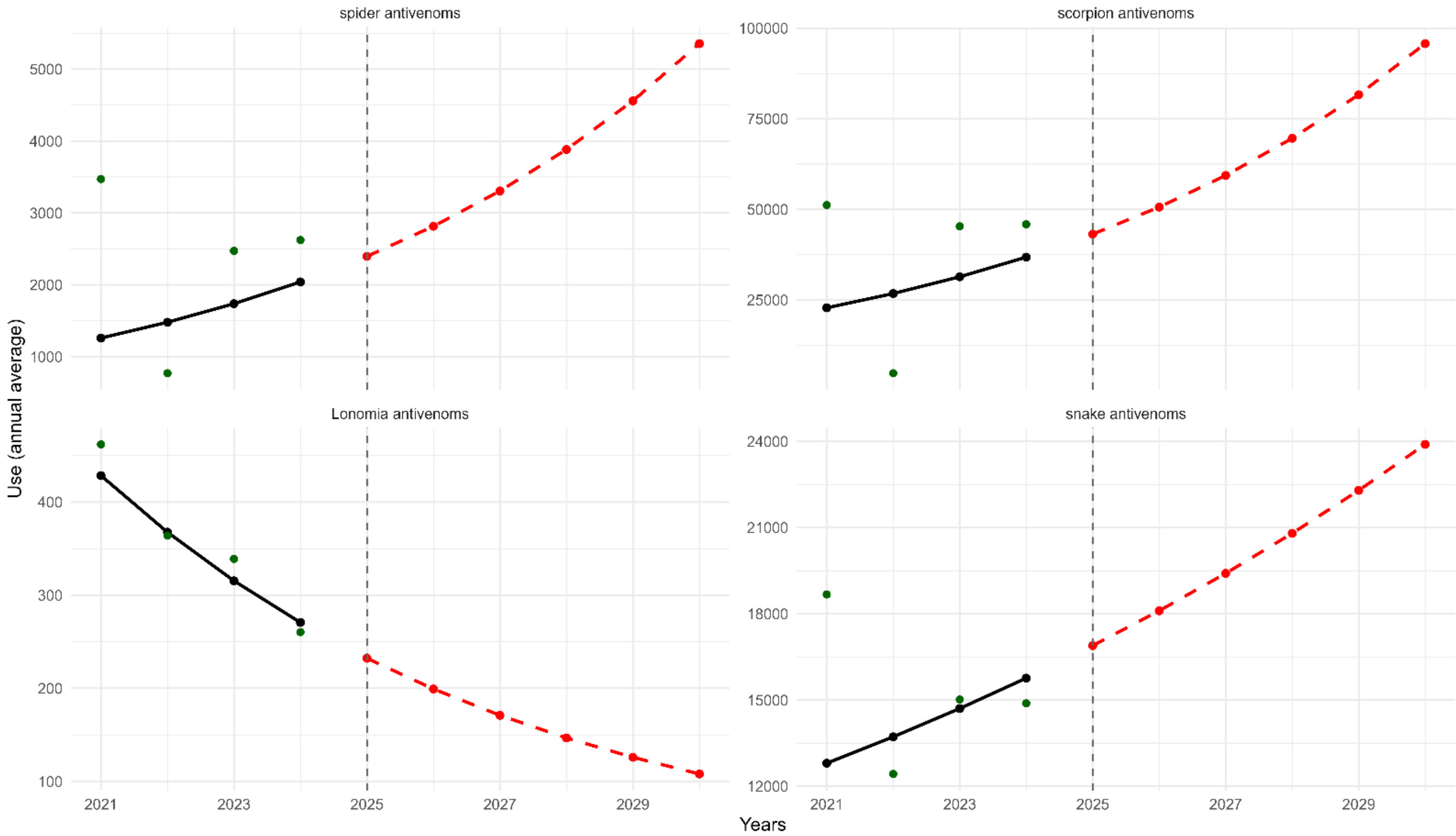

Figure S1. Temporal trend and prediction of the mean annual number of vials per type of antivenoms. The panels show the trend of the mean annual number of vials produced (**A**) by 12 Latin American public laboratories, procured (**B**) and used (**C**) within the healthcare system over the observed period (2021-2024; solid black lines) and the predicted period (2025-2030; dashed red lines), estimated from negative binomial models with population compensation. The 95% confidence intervals of the expected value (μ) were not represented due to the high variability among data. The dashed vertical line marks the beginning of the projected period. The green points represent the observed mean annual values, illustrating the substantial variation across years. It is important to note that there is also considerable within-year variability (although this is not shown in the figure), and that the mean values are largely influenced by the highest individual observations.
